# Altered mRNA Expression of Fucosyltransferases and Fucosidase Predicts Prognosis in Human Oral Carcinoma

**DOI:** 10.22088/IJMCM.BUMS.10.2.123

**Published:** 2021-09-01

**Authors:** Kruti Mehta, Kinjal Patel, Shashank Pandya, Prabhudas Patel

**Affiliations:** 1 *Molecular Oncology Laboratory, Cancer Biology Department, The Gujarat Cancer & Research Institute, Asarwa, Ahmedabad, Gujarat, India.*; 2 *Life Science Department, University School of Sciences, Gujarat University, Ahmedabad, Gujarat, India.*; 3 *Surgical Oncology Department, The Gujarat Cancer & Research Institute, Asarwa, Ahmedabad, Gujarat, India. *; 4 *Life Science Department, University School of Sciences, Gujarat University, Ahmedabad, Gujarat, India.*

**Keywords:** Oral cancer, hallmarks of cancer, glycosylation, fucosylation, fucosyltransferases, fucosidase

## Abstract

Aberrant protein glycosylation is known to be associated with the development of various cancers. Although fucosylation is essential for normal biological functions, alterations in fucosylation are strongly implicated in cancer and increasing metastatic potential. Altered fucosyltarnsferases (FUTs) and fucosidases are found to be involved in many types of malignancies. In this study, we examined the mRNA expressions of fucosidase (*FUCA1*) and FUTs (*FUT*s (*FUT3*, *FUT4*, *FUT5*, *FUT6*, *FUT8*) in human oral cancer tissues. All *FUT*s and *FUCA1* were significantly (P ≤0.05) down-regulated in malignant tissues in comparison with their adjacent normal tissues. The relationship between the clinicopathological parameters and the expression of *FUT*s and *FUCA1* revealed that higher mRNA levels of *FUT4*, *FUT5*, and *FUT8* and lower levels of *FUT3* were associated with progression of disease and lymph node metastasis in oral carcinoma indicating their role in oral cancer progression. Collectively, results suggest that elevated mRNA levels of *FUT4*, *FUT5* and *FUT8* may be used as worst prognostic indicators for oral carcinoma.

Glycosylation of proteins is the most important posttranslational modification, and altera-tions in patterns of glycosylation are known to occur during progression of cancer (1). Alterations in glycosylation influence various cellular functions including cell adhesion and cell immunogenicity, and also facilitate the generation of distant metastasis (2). Aberrant glycosylation is the result of alterations in genes which encode glycosylation enzymes, substrates, and donors. These effects predominantly occur from changes in gene expression levels of glycosyltransferases and glycosidases. Therefore, the analysis of genes involved in the glycosylation can assist in the discovery of molecular markers associated with the development and progression of several malignancies. Altered glycosylation has an important translational value as the distinctive alterations in tumor-associated glycosylation that provides a unique feature of cancer cells, and thus grant opportunities for novel diagnostic and even therapeutic targets. Earlier investigators have documented glycosylation as a new hallmark of cancer having a pivotal role in the development and progression of several malignancies (1, 3-4). Glycosylation depends on the action of glycosyltransferases and glycosidases in different tissues or cells (5). Sialylation and fucosylation are the major typical terminal modifications of proteins that mediate vital biological functions, and also have clinical implications in cancer (1, 6-7).

Fucosylation is one of the major branches of glycosylation. It transfers a fucose residue to oligosaccharides and proteins which are regulated by numerous enzymes like fucosyltransferases (FUTs), fucosidase and guanosinediphosphate (GDP)-fucose synthetic enzymes. Altered expression of various FUTs such as *FUT3* (8), *FUT4* (9), *FUT6* (10-11), *FUT7* (12-13), *FUT8* (14-15) mediate cancer cell migration, and thereby metastasis, suggesting that altered fucosylation may play an important role in disease progression. 

Another important enzyme component of fucosylation is α-L-fucosidase. α-L-fucosidase is a lysosomal enzyme that performs the hydrolytic cleavage of terminal fucose residue. The presence of fucosidases (FUCAs) is necessary for rapid turnover of N-glycans followed by reglycosylation and reinsertion of the proteins in plasma membrane (16). We previously reported serum α -L-fucosidase as a useful marker for monitoring oral cancer patients during their post–treatment follow-up (7). Our previous study has also reported significantly higher serum and salivary α-L-fucosidase activity in oral cancer patients as compared to controls (17). In the head and neck squamous cell carcinoma (HNSCC), primary tumors exhibiting higher FUCA1 expression are found to be significantly associated with worse patient survival (18).

Oral cancer is the highest occurring malignancy in India, harboring almost one third of the world burden of oral cancer (19). According to GLOBOCAN 2018, 1,19,992 new oral cancer cases and 72,616 deaths were reported from India with higher incidence in men compared to women (20). Management of oral carcinoma includes surgical resection and/or a combination of chemo- and radio-therapy (21-22). In India, majority of the oral cancer cases are diagnosed at advanced stages and have compromised treatment options. Hence, there is a vital need to discover newer drug targets and treatment modalities for the better management of oral cancer.

In the head and neck cancer, increased fucosylation has a crucial role in invasive and metastatic properties of the cancer stem cells (23, 24). Previously, we have reported higher fucosyltransferase and fucosidase activity in tissue, serum and saliva of oral cancer patients which revealed the importance of monitoring fucosylation changes during various stages of cancer progression which denotes its role in the early detection and better management of oral cancer patients (17, 7). However, to the best of our knowledge there are fewer studies presenting altered mRNA expression of fucosylation enzymes in oral cancer. Therefore, the present investigation was aimed to evaluate the clinical significance of mRNA expressions of various fucosyltransferses (*FUT3*, *FUT4*, *FUT5*, *FUT6*, *FUT8*) and fucosidases (*FUCA1*) in oral cancer progression. 

## Materials and methods

**Study subjects**

The study was approved by GCRI/GCS Ethics Committee (approval No. EC/08/2017 dated: 05/08/2017). 80 untreated oral cancer patients with no major disease in recent past were enrolled into the study. Oral cancer patients who have not undergone surgery, chemotherapy or radiotherapy in the past or at the time of enrollment were included in the study. The patients suffering from any other major illness in the past as well as HIV, HCV and/or HBV positive subjects were excluded from the study. Duly informed consent was obtained from the patients. The pathological staging pTNM-tumor, node, and metastasis of the patients were determined as per American Joint Committee on Cancer (AJCC) norms. The clinical details of the oral cancer patients are mentioned in Table 1. Majority of the patients were males (76.3%), tobacco habituates (97.3%), with tongue carcinoma as primary site (45%), early stage of the disease (48.7%) and moderate differentiation (66.3%).

**Sample collection**

80 malignant and their adjacent normal tissue samples were collected from oral cancer patients from operation theater immediately after surgical resection of the tumors, and were maintained on the ice. The tissue specimens were washed with ice-cold phosphate buffer saline (PBS: pH 7.4) and stored in RNA stabilizing agent (Ambion, USA) at -80 °C until analyzed.

**Gene expression analysis**

RNA was isolated from all tissue samples using RNA isolation kit (Qiagen, Germany) and stored at -80 °C until analysis. First-strand cDNA was obtained using the High capacity cDNA reverse transcription kit (Thermofisher Scientific, USA) by incubating 500 ng of total RNA in a final reaction volume of 25 µL, according to the manufacturer’s protocol on thermocycler (Proflex, Life Technologies). qPCR was performed using SYBR green mastermix (Qiagen, Germany) kit on Ariamax Real Time PCR instrument (AriaMx Real Time PCR, Agilent Technologies, USA) in 10 µl reaction volumes. Each reaction contained 4 ng cDNA, 5 µL SYBR Green PCR Master Mix, 0.7 µM forward and reverse primers. The reactions were incubated in a 96-well plate at 95 °C for 3 min, followed by 40 cycles of denaturation at 95 °C for 5 s and annealing for *FUT6*, *FUT8* and *FUCA1* at 60 °C for 1 min; *FUT3* at 65 °C for 30 s; *FUT4* at 55 °C for 1 min, and *FUT5* at 69 °C for 1 min. Relative gene expression was calculated by 2^-∆∆Ct ^method by using β-actin as a house-keeping gene. Amplification efficiency was calculated by serial dilution of pooled sample and matched with amplification efficiency of house-keeping gene. Duplicate analysis was carried out to calculate inter-assay and intra-assay variations. Table 2 shows the primer sequences.

**Statistical analysis**

Statistical analysis was carried out using SPSS statistical software version 21.0. Paired *t*-test was used to compare the mRNA levels of *FUT*s and fucosidase between adjacent normal and malignant tissues of the oral cancer patients. To analyze the association of various *FUT*s and fucosidase isoforms with clinico- pathological parameters, multivariate analysis with least significant difference (LSD) test was performed.

**Table 1:** Clinico-pathological details of the oral cancer patients

**Clinico-pathological details**	**Oral cancer patients** **(N=80)**	**Clinico-pathological details**	**Oral cancer patients** **(N=80)**
**Stage of the disease**		**Disease site**	
Early disease (I + II)	39 (48.7 %)	Buccal Mucosa	17 (21.3 %)
Advanced disease (III + IV)	32 (40.0 %)	Oral tongue	36 (45.0 %)
Unidentified	09 (11.3 %)	Others	27 (33.7 %)
**Tumor differentiation**		**Histopathology**	
Well	23 (28.7 %)	Squamous cell carcinoma	80 (100.0 %)
Moderate	53 (66.3 %)	**Lymph node metastasis**	
Poor	00	Yes	21 (26.3 %)
Unidentified	04 (5.0 %)	No	59 (73.7 %)

**Table 2:** Primer sequences and amplicon size

**Gene**	**Primer sequence**	**Amplicon size (bp)**
*FUT3*	F: 5′-AAGAAACACACAGCCACC-3′ R: 5′-AAGAAACACACAGCCACC-3′	191
*FUT4*	F: 5′-TCCTACGGAGAGGCTCAG-3′ R: 5′-TCCTCGTAGTCCAACACG-3′	134
*FUT5*	F: 5’-TGGGTGTGACCTCGGCGTGA-3’ R: 5’-AAACCAGCCTGCACCATCGCC-3’	129
*FUT6*	F: 5′-CATTTCTGCTGCCTCAGG-3′ R: 5′-GGGCAAGTCAGGCAACTC-3′	138
*FUT8*	F: 5’-AACTGGTTCAGCGGAGAATAAC-3’ R: 5’-TGAGATTCCAAGATGAGTGTTCG-3’	172
*FUCA1*	F: 5′-AGTCACCCTGTTGCCTATGG-3′ R: 5′-TTTGGCGCTTTTAGATTGCT-3′	190
β-actin	F: 5’ CATGTACGTTGCTATCCAGGC 3’ R: 5’ CTCCTTAATGTCACGCACGAT 3’	250

## Results

**Patients’ characteristics**

The cohort included 80 oral cancer patients with no prior treatment taken. The oral cancer patients` age ranged from 25-75 years with mean age of 45 years. Majority of the oral cancer patients 76.3 % (61/80) were males whereas 23.7% (19/80) were females. Out of the patients, 93.75 % (75/80) were tobacco users whereas only 6.25 % (5/80) had no history of tobacco use. Among the tobacco habituated cases, majority (41.25 %) were exclusively tobacco chewers, 21.25 % smokers, and the remaining 37.5 % showed mixed habit of tobacco chewing and smoking along with alcohol intake.

**Expression of various** ***FUT*** **s and fucosidase transcripts were lower in malignant tissues**

**Fig.1:**
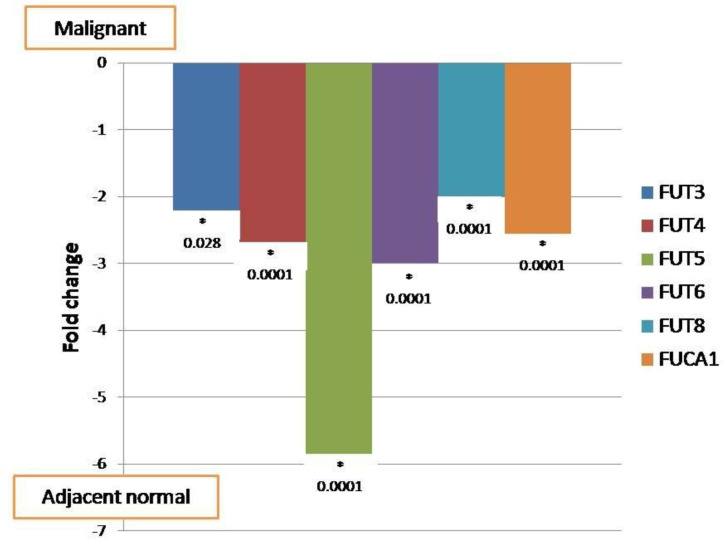
**Relative expression of** ***FUT*** **s and fucosidase transcripts in malignant vs. adjacent normal oral cancer tissues.** FUT= Fucosyltransferase

80 oral tumor tissues and their corresponding adjacent tissues were analyzed for mRNA expression of *FUT*s (*FUT3*, *FUT4*, *FUT5*, *FUT6*, *FUT8*) and fucosidase (*FUCA1*) by qRT-PCR. The specificity of the real time PCR was confirmed by melting curve analysis and agarose gel electrophoresis. Each transcript showed a theoretically expected single amplicon size in the electrophoresis (*FUT3*- 191 bp, *FUT4*- 134 bp, *FUT5*- 129 bp, *FUT6*- 138 bp, *FUT8*- 172 bp and *FUCA1*- 190 bp). Out of 80 oral cancer tissues, 27, 7, 17, 9, 3, 5 malignant tissue samples did not show expression of *FUT3*, *FUT4*, *FUT5*, *FUT6*, *FUT8*, and *FUCA1*, respectively. Our data demonstrated that tumor tissues contained significantly lower mRNA levels of *FUT3* (2.3 fold), *FUT4* (2.7 fold), *FUT5* (5.9 fold), *FUT6* (3.0 fold), *FUT8* (2.0 fold), and *FUCA1* (2.6 fold) as compared to adjacent normal tissues (Figure 1). 

**Differential expression of** ***FUT*** **s and** ***FUCA1*** **between tongue and buccal cancer**

**Fig.2:**
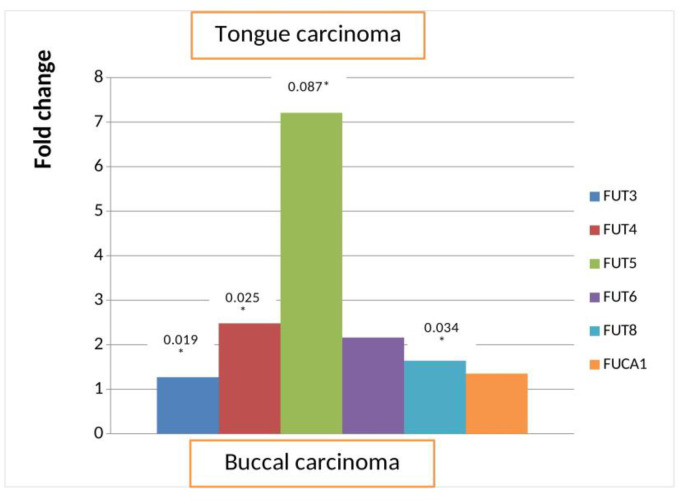
**Relative expression of FUTs and fucosidase transcripts in tongue vs. buccal mucosa as a primary site of the disease.** FUT= Fucosyltransferase

The majority of the patients involved in this study had tongue and buccal mucosa as a primary site of the disease. We compared the mRNA levels of *FUT*s and fucosidase between these two primary sites. There was a significant up regulation of *FUT3*, *FUT4*, *FUT5*, and *FUT8* (P = 0.019, 0.025, 0.087, and 0.034, respectively) in patients with tongue carcinoma in comparison with patients with buccal carcinoma as a primary site. Overall, mRNA levels of all *FUT*s and *FUCA1* were higher in tongue carcinoma patients in comparison with buccal carcinoma patients (Figure 2).

**Differential expression of** ***FUT*** **s and** ***FUCA1*** **among tobacco consumers in oral cancer patients**

**Fig.3:**
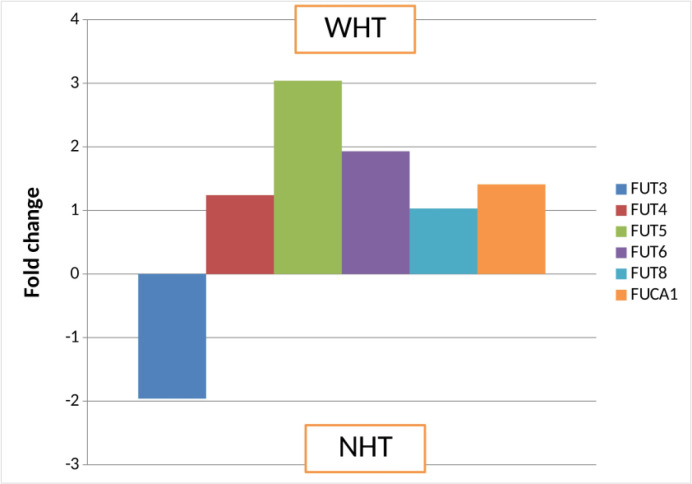
**Relative expression of** ***FUT*** **s and** ***FUCA1*** **transcripts in patients with tobacco habit (WHT) vs. patients without tobacco habit (NHT).** FUT= Fucosyltransferase

Tobacco consumption is one of the major risk factors for the development of oral cancer. To study the association between *FUT*s and *FUCA1* levels with tobacco habit, we compared the mRNA levels between the patients having tobacco habit (WHT, n=75) and patients who did not have tobacco consumption habit (NHT, n=5). Most interestingly, we found increased mRNA levels of *FUT5* (3.0 fold), *FUT6* (1.93 fold) and *FUCA1* (1.4 fold) in the patients having tobacco habit in comparison with the patients without tobacco habit (Figure 3).

**α 1-3/4, α 1-6 fucosyltransferase and fucosidase transcript levels were associated with clinicop-athological features of oral cancer patients**

**Fig.4:**
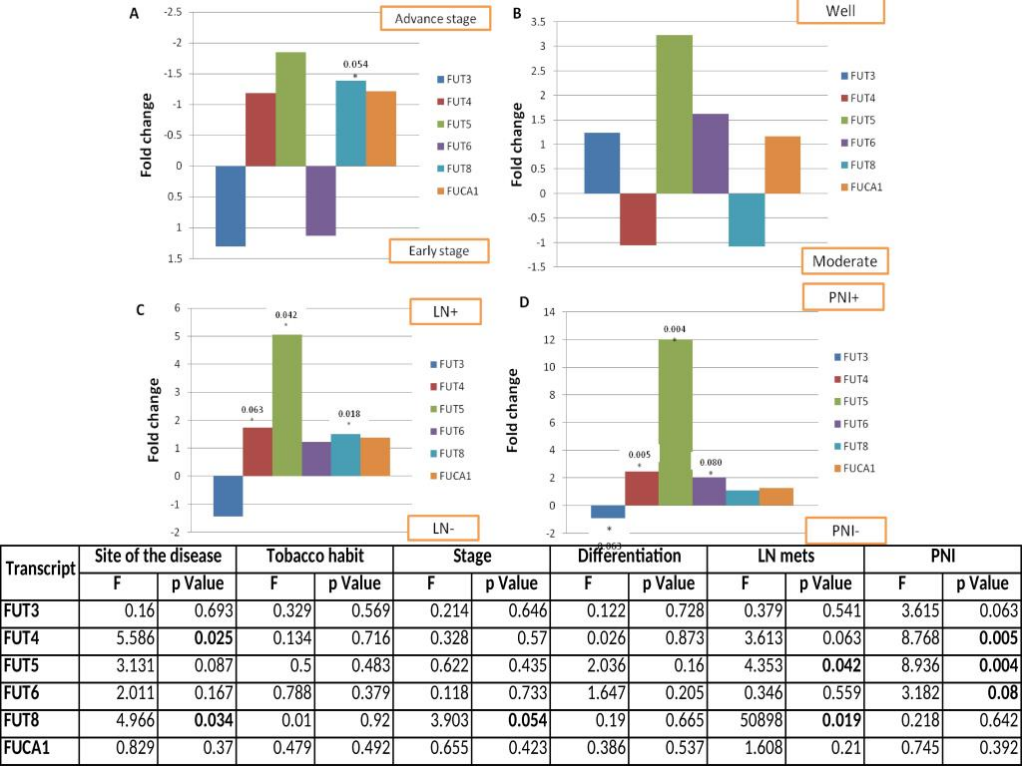
**Relative** **expressions of *FUT*s and *FUCA1* transcripts with respect to clinicopathological features.** A) Stage of the disease; B) Tumor differentiation; C) Lymph node status; D) Perineural invasion status. FUT: Fucosyltransferase; LN: Lymph node; PNI: Perineural invasion

There was a significant inter-correlation between all fucosyltransferases and fucosidase expression (Fig. 4). On multivariate analysis, it was observed that *FUT8* was significantly up regulated 1.4 fold (P = 0.054) and *FUT5* mRNA level also increased 1.8 fold in the advanced stage of the disease as compared to the early stage of the disease (Figure 4A). Furthermore, we correlated the transcripts levels with tumor differentiation. *FUT3*, *FUT5*, *FUT6*, and *FUCA1* mRNA levels were higher (1.2, 3.2, 1.6, and 1.2 log fold, respectively) in well-differentiated tumors as compared to moderately differentiated tumors (Figure 4B). Regional lymph node metastasis and perineural invasion (PNI) are the major events involved in the loco-regional recurrence of the oral carcinoma. Therefore, we evaluated the association of mRNA levels of *FUT*s and *FUCA1* with lymph node positive patients. We found significant up regulation of *FUT5* and *FUT8* (P = 0.042 and 0.019, respectively) whereas 1.7 fold up regulation of *FUT4* was observed in the lymph node positive patients as compared to the lymph node negative patients. *FUT3* mRNA levels were also found to be 1.4 fold decrease in the patients having LN^+^ status as compared to the patients having LN^- ^status (Figure 4C). Further, we observed significant up regulation of *FUT4* (2.5 fold, P = 0.005) and *FUT5* (12.0 fold, P = 0.004), and 3.3 fold down regulation of *FUT3* in patients who had positive perineural invasion status as compared to patients with negative perineural invasion status. *FUT6* levels were also 2.0 fold increase in the PNI^+ ^patients as compared to PNI^-^ patients (Figure 4D).

All together from this study, multivariate analysis revealed that *FUT4* and *FUT5* were upregulated whereas *FUT3* was downregulated with disease advancement, in patients with lymph node positive and perineural invasion positive status. Increased *FUT4* and *FUT5* levels were also observed in tongue carcinoma which is a more aggressive form of oral cancer, and also in the patients having tobacco consumption habit. 

## Discussion

In this study, significant down regulation of *FUT*s and *FUCA1* was observed in oral cancer tissues in comparison with adjacent normal tissues. Similar observations were reported in patients with colorectal adenocarcinoma showing significant decrease in *FUCA1* activity in malignant tissue compared to healthy colonic mucosa in the same patients (25). In the present investigation, down regulation of all *FUT*s can be attributed to the “adjacent normal” part of the tissue, which is actually pathologically normal but may have certain cells bearing the early changes of malignant transformation known as field cancerization. Also, in the majority of oral cancer patients, the adjacent normal tissue has very close proximity to the tumor part, which leads to the early changes in the normal glycosylation patterns in non-malignant part of oral cavity.

Several authors have reported that alteration in fucosyltransferase activities and fucosylation levels have significant role in the tumor progression and metastasis in various malignancies (13, 23, 26-28). Therefore, we analyzed the association between mRNA levels of *FUT*s and *FUCA1* with various clinico-pathological parameters of oral cancer patients. Increased mRNA levels of *FUT3*, *FUT5*, *FUT6* and *FUCA1* were observed in well-differentiated tumors as compared to moderately differentiated tumors. Cheng et al. (29) have suggested that high *FUCA1* expression can alter the composition and decrease the quantity of cell surface fucosylation associated molecules, thereby limiting the invasiveness of cancer cells in the early-stage breast tumors. In another study, colorectal adenocarcinoma patients showed significant decrease in *FUCA1* activity with the progression of the disease from early to advanced stages (30). Moreover, significant up regulation of *FUT8* (P = 0.054) and 1.85 fold up regulation of *FUT5* mRNA levels were observed in tumor with advanced stage as compared to tumor with early stage. These data suggest that increased mRNA levels of *FUT8* and *FUT5* can be associated with tumor progression in oral carcinoma. Similarly, Liang et al. (31) have reported that the higher expression of *FUT5* in CRC tissues and cell lines, showed enhanced proliferation, migration, invasion, and angiogenesis capacity of CRC cells and tumour growth *in vivo*. 

Furthermore, we compared the mRNA levels between LN^+^ and PNI^+^ tumors, and LN^-^ and PNI^-^ tumors. In the LN^+^ patients, significant up regulation of *FUT5* and *FUT8* as well as the increased levels of *FUT4* was observed as compared to LN^-^ patients. Significant up regulation of *FUT4* and *FUT5* whereas down regulation of *FUT3* (3.37 fold) was observed in the PNI^+^ cases as compared to the PNI^-^ cases. Zhan et al. (32) have reported that the knockdown of *FUT3* results into the inhibition of the tumorigenesis *in vivo*, which provides a promising target for reducing the metastatic virulence of pancreatic cancer cells. In our data, increased level of *FUT6* (2.0 fold) was also observed in patients having PNI^+^ status as compared to PNI^-^ patients. In line with our data, higher *FUT8* protein expression was found to be correlated with lymphatic metastasis in breast carcinoma tissues (33). In melanoma, up regulation of *FUT8* was identified as a driving factor of metastasis via reducing cleavage of fucosylated adhesion molecule, L1CAM (34-35). Furthermore, higher expression of *FUT8* was also found to be associated with larger tumor size and lymph node metastasis in papillary thyroid carcinoma (36). Over expression of *FUT8* was also found to be correlated with increased fucosylation of glycoproteins in aggressive prostate cancer cells (37). However, to the best of our knowledge, no such studies have extensively analyzed the role of *FUT*s and *FUCA1* in oral cancer progression. 

In the present study, most patients had buccal carcinoma and tongue carcinoma as the primary site of the disease. Analyzing the mRNA expression of FUTs and *FUCA1* revealed that, mRNA levels of FUTs and *FUCA1* were higher with significant up regulation of *FUT4* and *FUT8* in the tongue carcinoma patients as compared to the buccal carcinoma as a primary site of the disease. Tongue carcinoma is known to be more aggressive because of its close proximity to major nerves and neck nodes, and also has a rich blood supply. In the present study, we observed higher increased fucosylation transcripts in tongue carcinoma which suggests its potential role in disease aggressiveness. Furthermore, the expression of *FUT4*, *FUT5*, *FUT6* and *FUCA1* were higher whereas *FUT3* levels were lower in patients with tobacco consumption habit in comparison with patients without tobacco habit. The majority of the patients in the present study had tobacco habits in the form of tobacco smoking and chewing. The smokeless tobacco as well as cigarette smoking leads to the formation of bulky DNA adducts. The bulky DNA adducts cause permanent cellular mutations leading to activation of oncogenes and inactivation of tumor suppressor genes, resulting in neoplastic transformation of normal cells (38). Further, we have documented that the treatment of tobacco metabolites such as 4-NQO, NNK, Benzopyrene lead to the increase in *FUT4, FUT5, FUT6*, and *FUCA1* transcripts in a dose dependent manner in treated tongue carcinoma cells (39). Thus, it can be assumed that tobacco metabolites play a key role in the progression of oral cancer by altering the normal glycosylation pattern leading to the worsening of the disease 

progression. 

To the best of our knowledge, this is the first study of its kind that has analyzed the tissue mRNA levels of various *FUT*s and *FUCA1* in the association with disease progression and loco-regional recurrence in oral cancer patients. The present study documented the higher mRNA levels of *FUT4*, *FUT5*, and *FUT8* in advanced stage and metastatic tumor which highlights their role as valuable indicators of aggressiveness and metastatic potential of the disease. Further, down regulation of *FUT3* with disease advancement suggests its role as a good prognosticator for oral carcinoma. Hence, these molecular signatures open the possibility of new therapeutic opportunities for targeting highly metastatic oral carcinoma by several strategies like nutraceutical therapies alone or in combination with other conventional therapies with the use of molecules targeting aberrant fucosylation. Further, the results strongly warrant evaluation of epigenetic regulation of fucosylation transcripts to unravel the molecular mechanisms behind aberrant fucosylation in oral carcinoma. 
